# Assessing the causal association of trauma with subsequent psychiatric disorders by a Mendelian randomization study trauma and common psychiatric disorders

**DOI:** 10.3389/fpsyt.2023.1152005

**Published:** 2023-07-24

**Authors:** Dongqing Gu, Shan Ou, Guodong Liu

**Affiliations:** ^1^Department of Epidemiology and Biostatistics, First Affiliated Hospital, Army Medical University, Chongqing, China; ^2^Department of Anesthesiology, First People’s Hospital of Chengdu, Chengdu, China; ^3^Department of Wound Care Support, State Key Laboratory of Trauma, Burns and Combined Injury, Research Institute of Surgery, Daping Hospital, Army Medical University, Chongqing, China

**Keywords:** trauma, psychiatric disorders, depression, Mendelian randomization, causal association

## Abstract

**Objective:**

Trauma has been proposed as a risk factor for the development of psychiatric disorders. This study aimed to determine the causal role of trauma in six common psychiatric disorders.

**Methods:**

We obtained summary-level data for genetic variants associated with trauma and the corresponding association with psychiatric disorders from previous genome-wide association studies. Two-sample Mendelian randomization analyzes were performed to estimate the causal association between trauma and psychiatric disorders, with inverse variance weighted used as the main method.

**Results:**

Genetically predisposed trauma was associated with an increased risk of psychiatric disorders [odds ratio (OR) =1.24, 95%, confidence interval (CI), 1.09–1.40], anxiety disorder (OR = 1.30, 95% CI, 1.10–1.52) and schizophrenia (OR = 1.48, 95% CI, 1.18–1.84). However, the associations between trauma and sleep disorder (OR = 1.17, 95% CI, 1.01–1.35), as well as depression (OR = 1.09, 95% CI, 1.02–1.16) did not reach a Bonferroni corrected significance level. Besides, no association was observed between trauma and risk of bipolar disorder (OR = 1.21, 95% CI, 0.98–1.48) and eating disorder (OR = 1.28, 95% CI, 0.88–1.86).

**Conclusion:**

Trauma might be causally associated with an increased risk of some common psychiatric disorders such as anxiety disorder and schizophrenia. However, little evidence supported an association between trauma and risk of depression, bipolar disorder, sleep disorder, and eating disorder. Our findings offered novel insights into the trauma-mediated development mechanism of psychiatric disorders, and psychological intervention to patients with trauma may be an effective prevention strategy for psychological diseases.

## 1. Introduction

An estimated 89% of city dwellers have experienced traumatic events (1). Experiencing traumatic events mainly include exposure to childhood physical, sexual, and emotional abuse; partner-based physical, sexual, and emotional abuse and other traumatic events, including exposure to sexual assault, violent crime, life-threatening accident, and witnessing violent death. Several studies reported trauma exposure may contribute to subsequent occurrence of psychiatric disorders, such as depression (2, 3), schizophrenia (4), bipolar disorder (5), anxiety disorder (3), sleep disorder (6), and eating disorder (7, 8). Since the results of observational studies are usually interfered by confounding factors, whether the relationship between traumatic events and psychiatric disorders is causal still remains unclear (9). Randomized clinical trial (RCT) is arguably the gold standard to infer causality, while it might be prohibitively costly and require consideration of ethics, or be otherwise unfeasible.

A complement to traditional epidemiology is to conduct Mendelian randomization (MR) analysis. This method rests on three main assumptions: assumption one is that the genotype must be associated with the phenotype, assumption two is that the genotype should not be associated with confounders, assumption three is that the genotype should affect the outcome only through the risk factor. It utilizes genetic variants as a proxy for an exposure of interest to investigate its potential to have a causal association with a disorder (10). As the inheritance of genetic variants is randomized at meiosis, the association between the alleles and the outcome are unlikely to be affected by reverse causality and confounding factors (11). Importantly, the available studies have demonstrated that reported trauma exposure is heritable, with twin heritability estimates of 20–50% (12, 13), and single nucleotide polymorphisms (SNPs)-based heritability estimates of 30% (14). Recently, a genome-wide association study (GWAS) reported that SNPs were associated with trauma, making it possible to explore the causal relationship between trauma and psychiatric disorders (15). Using MR method, trauma exposure was demonstrated to increase the risk of major depressive disorder (16).

In this study, we used a two-sample Mendelian randomization approach to comprehensively estimate genetically predisposed trauma and risk of overall and six common psychiatric disorders (including depression, bipolar disorder, sleep disorder, schizophrenia, anxiety disorder, and eating disorder) using summary data from the previous GWAS. This study aims to shed light on whether trauma was a causal risk factor of psychiatric disorders.

## 2. Materials and methods

### 2.1. Data source

Summary statistics for estimating the genetic association of trauma and psychiatric disorders were obtained from GWAS catalog,^1^ and MR-Base platform.^2^ The detail information of data source was shown in Table 1. In detail, the summary statistics for trauma were derived from UK Biobank including 98,720 European ancestry participants (35,269 cases and 63,451 controls) (15). The summary statistics for overall psychiatric disorders (54,496 cases and 164,296 controls), anxiety disorder (20,992 cases and 197,800 controls), sleep disorder (19,155 cases and 197,545 controls) and eating disorder (1,874 cases and 216,918 controls) were derived from FinnGen. The summary statistics for depression were derived from PGC, UK Biobank, GERA cohort including 180,866 European ancestry participants (105,739 cases and 16,471 controls) (17). The summary statistics for bipolar disorder were derived from Bipolar Disorder Working Group of PGC including 51,710 European ancestry participants (20,352 cases and 31,358 controls) (18). The summary statistics for schizophrenia were derived from Schizophrenia Working Group of PGC including 77,096 European ancestry participants (33,640 cases and 43,456 controls) (19).

**Table 1:** Details of the data sources used in this study.

Traits	Definitions	Number of cases	Number of controls	Sample size	Consortium or cohorts	PubMed ID or web-link	Exposure/Outcome
Trauma	Trauma exposure mainly included three categories (child trauma, adult trauma, PTSD-relevant trauma)	35,269	63,451	98,720	UK Biobank	31,969,693	Exposure
Psychiatric disorders	-	54,496	164,296	218,792	FinnGen	https://www.finngen.fi/fi	Outcome
Depression	Depressive symptoms	105,739	16,471	180,866	PGC, UK Biobank, GERA cohort	27,089,181	Outcome
Bipolar disorder	International consensus criteria (DSM-IV, ICD-9, or ICD-10) for a lifetime diagnosis of BD established using structured diagnostic instruments from assessments by trained interviewers, clinician-administered checklists, or medical record review	20,352	31,358	51,710	Bipolar Disorder Working Group of PGC	31,043,756	Outcome
Anxiety disorder	Main ICD10	20,992	197,800	218,792	FinnGen	https://gwas.mrcieu.ac.uk/	Outcome
Sleep disorder	–	19,155	197,545	216,700	FinnGen	https://www.finngen.fi/fi	Outcome
Eating disorder	–	1874	216,918	218,792	FinnGen	https://www.finngen.fi/fi	Outcome
Schizophrenia	Schizophrenia is characterized by psychosis and negative symptoms such as social and emotional withdrawal	33,640	43,456	77,096	Schizophrenia Working Group of PGC	25,056,061	Outcome

BD, Bipolar disorder; PGC, Psychiatric Genomics Consortium (PGC); PTSD, Post-Traumatic Stress Disorder.

### 2.2. Selection of genetic variants

Trauma exposure mainly included three categories: child trauma (22,490 individuals), adult trauma (30,695 individuals), post-traumatic stress disorder (PTSD-relevant trauma) (18,397 individuals) according to the recent published GWAS. It was defined as report at least two of the following items: felt loved as a child less than “often,” felt hated by a family member as a child more than “never,” sexually abused as a child more than “never,” physical violence by a partner more than “never,” belittlement by a partner more than “never,” sexual interference by a partner more than never, and victim of sexual assault (15). SNPs associated with trauma were selected at the *p*-value <5 × 10^−6^, since only several SNPs arrived at a genome-wide significance level (*p* < 5 × 10^−8^). After extracting the summary data for the SNPs, we pruned all SNPs in linkage disequilibrium (LD) in LDlink^3^ (r^2^ threshold <0.01), and selected the SNPs with the lowest *p*-value as an independent instrument, resulting in a total of 60 independent SNPs associated with trauma. We calculated the proportion of variance (R^2^) explained in the risk factor by the SNP(s) and the strength of the instrument (*F*-statistic) using the formulae from Yarmolinsky et al. (20). The variance explained by the independent variants was 1.48%, and the *F*-statistic was 25, satisfying the threshold of >10 (21). Detailed information of the SNPs used as instrumental variables was displayed in Supplementary Table S1.

### 2.3. Mendelian randomization analysis

The inverse variance weighted (IVW) method was used to estimate the effect of the exposure on the outcome from the slope of the relationship between bXG (SNP-exposure association) and bYG (SNP-outcome association). Sensitivity analyzes were performed using several other methods (maximum likelihood, MR-Egger, and weighted median) to evaluate the robustness of our main analyzes. Leave-one-out analysis was employed to test the sensitivity of our results to single SNP effects. To further assess the potential presence of horizontal pleiotropy, we used Cochran’s Q for heterogeneity and the intercept from the MR-Egger method (22, 23).

### 2.4. Statistical analysis

All analyzes were conducted using the package TwoSampleMR (version 0.5.6) in R (version 4.1.2), and the TwoSampleMR R package curated by MR-Base. All tests were two-sided, and a Bonferroni corrected significance level of *p* < 0.006 (0.05/8) was used. In addition, we performed a power calculation using PASS 2019 (v19.0.2). Given our sample size (the minimum sample size was 51,710), we had 100% power to detect a minimal odds ratio (OR) of 1.10 at a statistical significance level of 0.05.

## 3. Results

**Figure 1:**
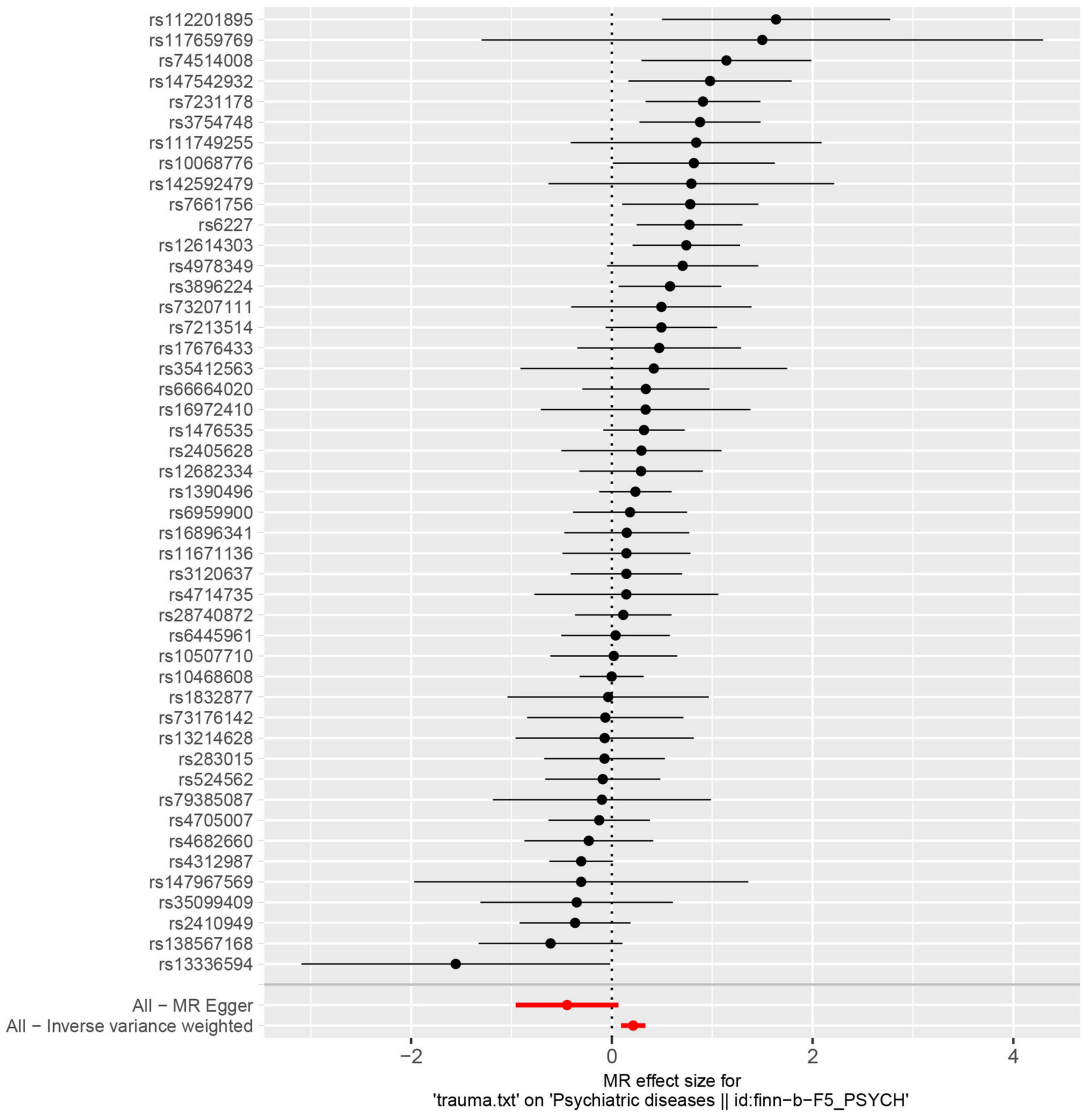
Forest plot of the genetic causal association between trauma and psychiatric disorders.

Genetically predisposed trauma was associated with an increased risk of overall psychiatric disorders [OR = 1.24, 95%, confidence interval (CI), 1.09–1.40; *p* = 0.0006], as well as anxiety disorder (OR = 1.30, 95% CI, 1.10–1.52; *p* = 0.002) and schizophrenia (OR = 1.48, 95% CI, 1.18–1.84; *p* = 0.0005). However, the associations between trauma and sleep disorder (OR = 1.17, 95% CI, 1.01–1.35; *p* = 0.04), as well as depression (OR = 1.09, 95% CI, 1.02–1.16; *p* = 0.01) did not reach a Bonferroni corrected significance level. Besides, no association was observed between genetically predisposed trauma and risk of bipolar disorder (OR = 1.21, 95% CI, 0.98–1.48; *p* = 0.07) and eating disorder (OR = 1.28, 95% CI, 0.88–1.86; *p* = 0.19) (see Table 2 and Figure 1).

**Table 2:** Mendelian randomization analyzes of the causality between genetically predisposed trauma and psychiatric disorders risk.

Outcome	Methods	SNPs (N)	OR (95% CI)	*p* value	Heterogeneity Q tests *P* value	MR-Egger intercept test *p* value
Psychiatric disorders	IVW	47	1.24 (1.09–1.40)	0.0006	0.0009	0.01
	Maximum likelihood		1.26 (1.14–1.38)	4.69E-06		
	MR Egger		0.64 (0.37–1.08)	0.10		
	Weighted median		1.16 (0.99–1.34)	0.07		
Depression	IVW	37	1.09 (1.02–1.16)	0.01	5.963E-7	0.31
	Maximum likelihood		1.10 (1.05–1.16)	4.56E-05		
	MR Egger		0.94 (0.70–1.26)	0.66		
	Weighted median		1.04 (0.96–1.12)	0.37		
Bipolar disorder	IVW	47	1.21 (0.98–1.48)	0.07	0.003	0.13
	Maximum likelihood		1.21 (1.03–1.42)	0.02		
	MR Egger		0.60 (0.23–1.51)	0.27		
	Weighted median		1.14 (0.89–1.48)	0.30		
Anxiety disorder	IVW	47	1.30 (1.10–1.52)	0.002	0.003	0.02
	Maximum likelihood		1.30 (1.14–1.48)	8.91E-05		
	MR Egger		0.58 (0.29–1.16)	0.13		
	Weighted median		1.17 (0.96–1.42)	0.12		
Sleep disorder	IVW	47	1.17 (1.01–1.35)	0.04	0.08	0.14
	Maximum likelihood		1.17 (1.02–1.34)	0.02		
	MR Egger		0.72 (0.37–1.39)	0.33		
	Weighted median		1.17 (0.88–1.31)	0.50		
Eating disorder	IVW	47	1.28 (0.88–1.86)	0.19	0.45	0.73
	Maximum likelihood		1.29 (0.88–1.88)	0.19		
	MR Egger		1.71 (0.31–9.38)	0.54		
	Weighted median		1.10 (0.62–1.96)	0.75		
Schizophrenia	IVW	45	1.48 (1.18–1.84)	0.0005	4.982E-12	0.02
	Maximum likelihood		1.56 (1.34–1.80)	4.03E-09		
	MR Egger		0.48 (0.19–1.23)	0.13		
	Weighted median		1.31 (1.03–1.65)	0.02		

CI, Confidence interval; IVW, Inverse variance weighted; OR, Odds ratio; SNP, Single nucleotide polymorphism.

**Figure 2:**
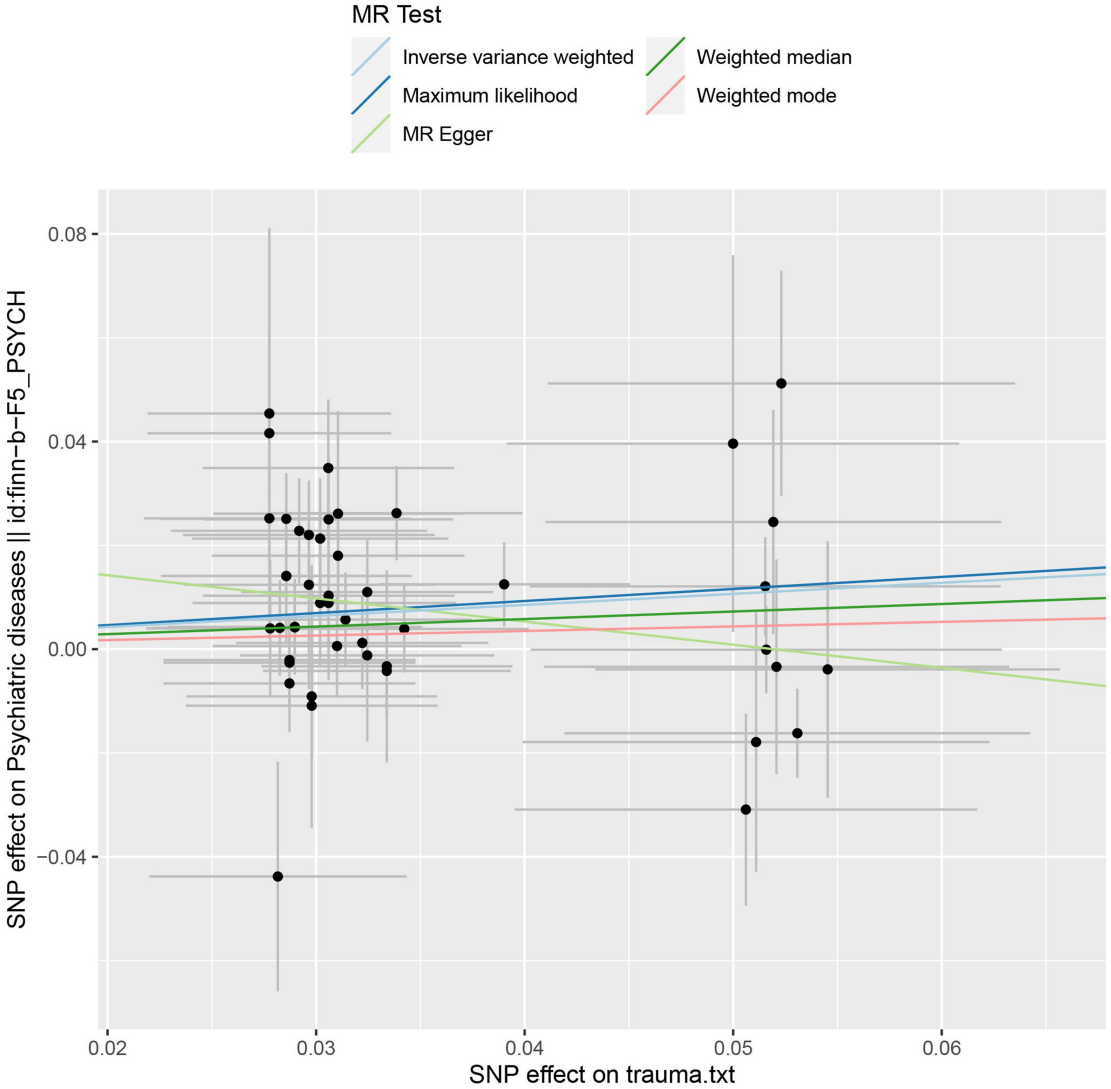
Scatter plot of the genetic causal association between trauma and psychiatric disorders.

**Figure 3:**
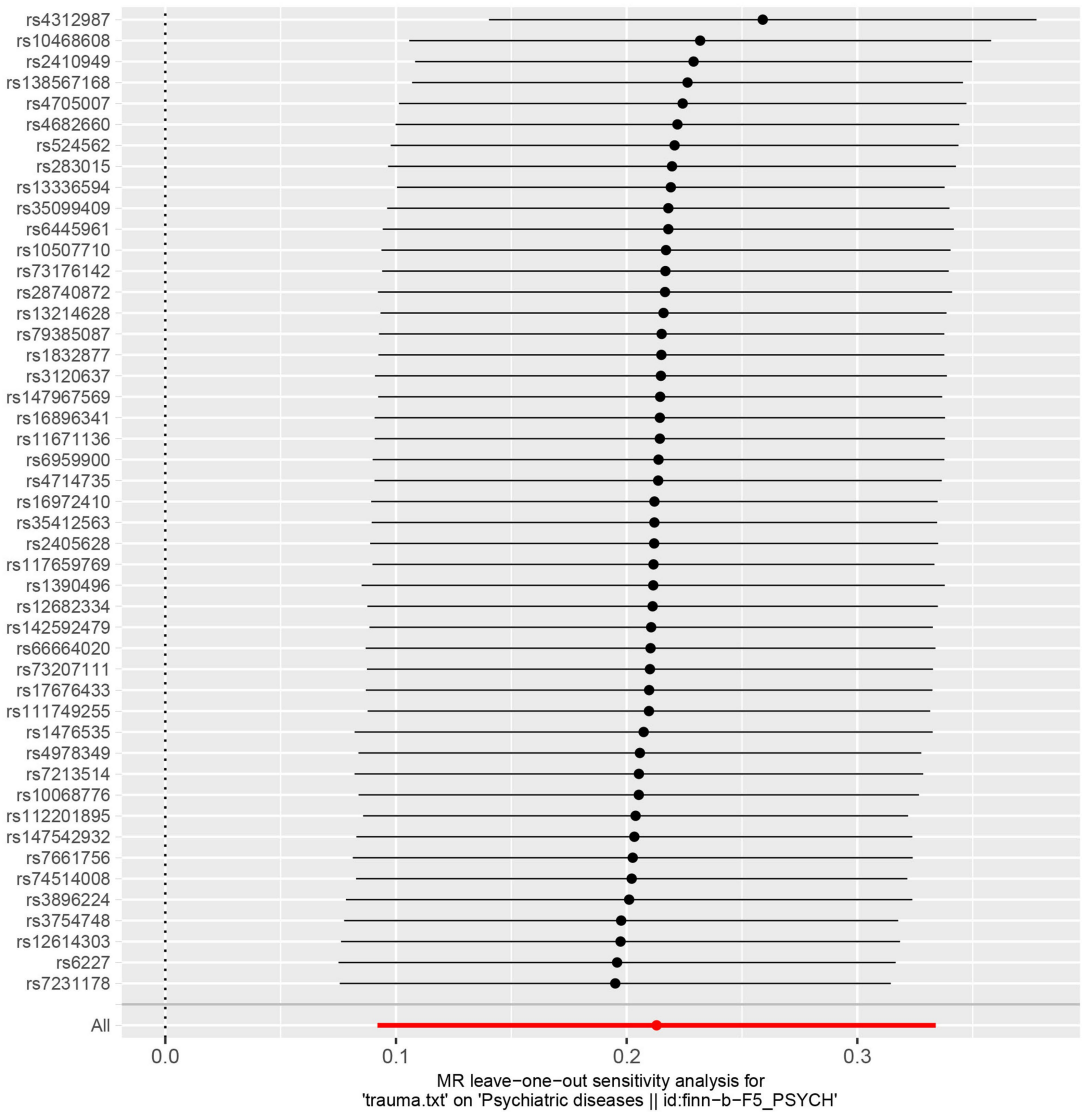
Leave-one-out plot of the genetic causal association between trauma and psychiatric disorders.

**Figure 4:**
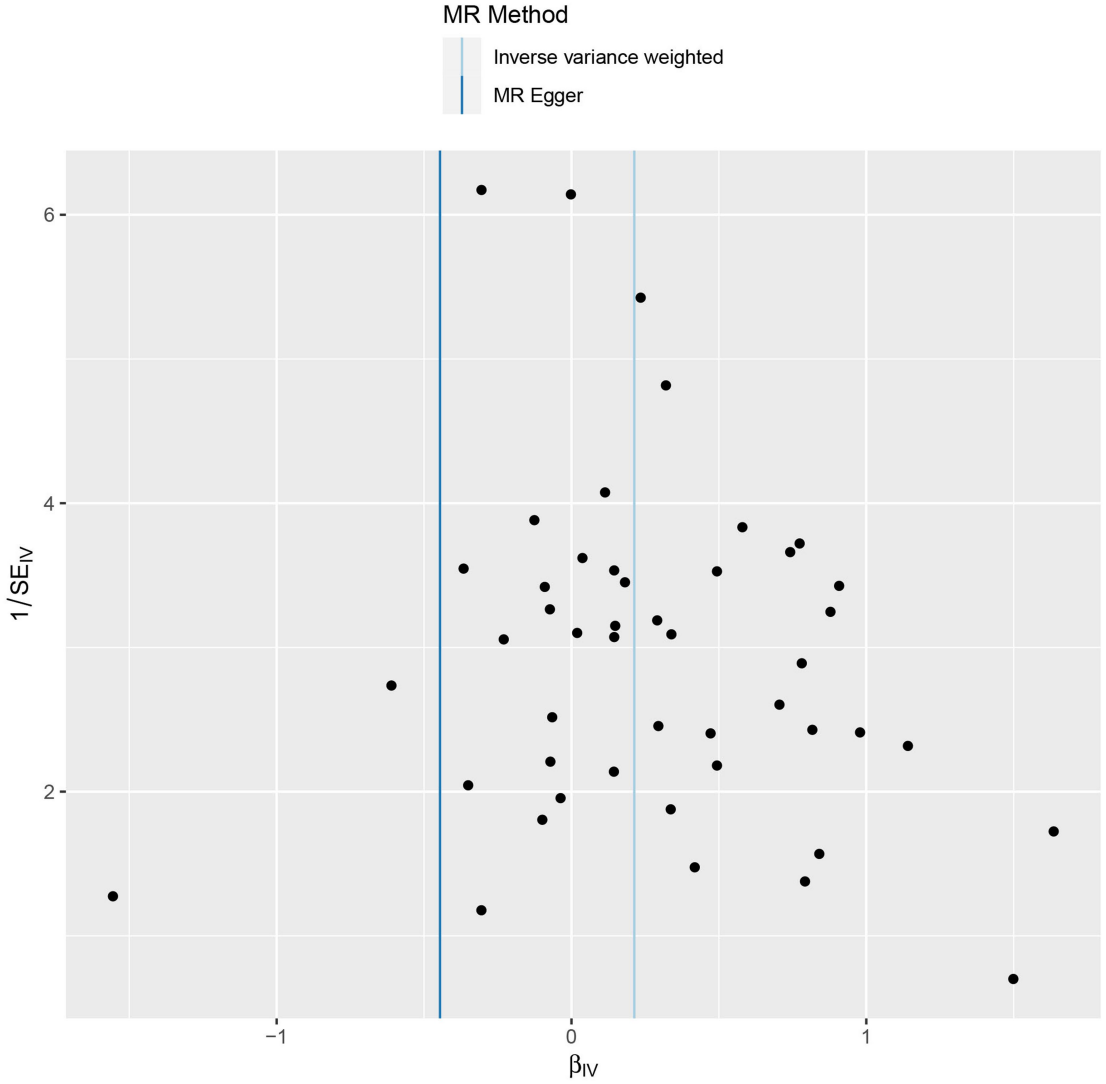
Funnel plot of the genetic causal association between trauma and psychiatric disorders.

Sensitivity analyzes were performed using different Mendelian randomization methods, and the results showed these findings were partly consistent (see Table 2 and Figure 2). In addition, “leave-one-out” analysis demonstrated that no single instrument included strongly affected the outcomes of above Mendelian randomization analysis (see Figure 3). Finally, the funnel plot showed that none of the included IVs had a potential bias, indicating stable results in the study (see Figure 4).

Heterogeneity and horizontal pleiotropy were estimated in this study, however, significant heterogeneities (Heterogeneity Q tests *p* < 0.05) were observed for most of the associations, and horizontal pleiotropy (MR-Egger intercept test *p* < 0.05) was also observed for associations between trauma and anxiety disorder and schizophrenia (see Table 2).

## 4. Discussion

In this study, we performed a two-sample Mendelian randomization analysis using trauma-associated SNPs as genetic instruments to determine the causal role of trauma in risk of overall as well as six common psychiatric disorders. Our study suggested that trauma exposure was causally associated with an increased risk of psychiatric disorders, anxiety disorder and schizophrenia, which highlighted the critical role of trauma exposure in shaping risk for psychopathology throughout the life course. This study may be useful in providing new insights into the development mechanism of trauma-mediated psychiatric disorders, therefore, psychological intervention to patients with trauma may be an effective prevention strategy for psychological diseases.

Mendelian randomization analysis rests on three main assumptions (24). Assumption one is that the instrumental variables must be associated with the phenotype. To satisfy the first assumption, we selected SNPs with a genome-wide significant association with trauma (15). Assumption two is that the instrumental variables should not be associated with confounders. Assumption three is that the instrumental variables should affect the outcome only through the phenotype. Therefore, we carried out statistical tests and sensitivity analyzes to evaluate the potential violation of the second and third assumptions. We evaluated horizontal pleiotropy by MR-Egger regression method, whereas we found the presence of horizontal pleiotropy in some of the reported associations. Psychiatric disorders were reported to share common variant risk, suggesting substantial pleiotropy of contributing loci (25). Therefore, we speculate there are shared genetic risks between exposure to potentially traumatic events and psychiatric conditions.

Although trauma has been shown to play a meaningful role in the etiology of some common psychiatric disorders, the associations were weak, and the relationship between them is complex and poorly understood. Previous studies have provided evidence for underlying mechanisms through which trauma may influence mental health. The hypothalamic–pituitary–adrenal (HPA) axis is a key regulator of the response to stress, and HPA axis dysregulation has been reported in various psychiatric disorders (26). Specially, it was suggested that HPA axis dysregulation may be the basis for an important etiological link between trauma exposure and subsequent psychiatric disorders (27, 28). However, the relationship remains complex and warrants further investigation. In addition, trauma appears to be an important correlate of a cascade of psychiatric disorders, between which both gene–environment correlations and gene–environment interactions have been observed (15, 29). Further studies are needed to illustrate the possible mechanisms.

Our study has several limitations. First, although the *F*-statistic satisfied the threshold, the selected SNPs for trauma exposure could only explain 1.79% of the phenotypic variation, other unknown trauma-related SNPs could also play an important role in the development of psychiatric disorders. Second, some traits in this study are vague terms and usually defined with different criteria across different studies, like depression and eating disorder, and there are also overlaps between these traits. Third, statistical heterogeneity was observed in some estimates, which may require further investigation. Fourth, Since the Psychiatric Genomics Consortium (PGC) GWAS contained participants from UK Biobank, the overlap between samples may exist. However, we are unable to quantify as individual-level data is not available for all individuals in the PGC GWAS. Fifthly, the MR Egger estimates are all quite different from other MR methods, and the horizontal pleiotropy may contribute to the bias of the results. Nevertheless, the results of the other three MR methods are consistent. Finally, the summary statistics used in our study were from participants of European ancestry, which limits the inference of findings in other populations, further studies in non-European populations are required.

## 5. Conclusion

In summary, our study suggested a causal effect of trauma on several common psychiatric disorders, however, the evidence was weak. Additionally, the results did not support an association between trauma and risk of depression, bipolar disorder, sleep disorder, or eating disorder. Better designed cohort studies and MR analysis are necessary to examine our findings, and deepen our understanding of their associations.

## Data availability statement

The original contributions presented in the study are included in the article/Supplementary material, further inquiries can be directed to the corresponding author.

## Author contributions

GL and DG contributed to conception and design of the study. DG and SO did the literature search, data extraction, and quality assessment. GL and DG did statistical analysis. DG wrote the first draft of the original manuscript with significant contributions from SO and GL. All authors contributed to the article and approved the submitted version.

## Funding

This study was funded by National Natural Science Foundation of China (81903393), the Military Biosafety Project (no.19SWAQ18), and Special Program (2022-JCJQ-ZD-224-12). The sponsors of this study had no role in study design, data collection, analysis, interpretation, writing of the report, or the decision for submission.

## Conflict of interest

The authors declare that the research was conducted in the absence of any commercial or financial relationships that could be construed as a potential conflict of interest.

## Publisher’s note

All claims expressed in this article are solely those of the authors and do not necessarily represent those of their affiliated organizations, or those of the publisher, the editors and the reviewers. Any product that may be evaluated in this article, or claim that may be made by its manufacturer, is not guaranteed or endorsed by the publisher.

## Footnotes

^1^ https://www.ebi.ac.uk/gwas/

^2^ https://www.www.mrbase.org

^3^ https://ldlink.nci.nih.gov
