# Supplementary material for: Assessing the causal association of trauma with subsequent psychiatric disorders by a Mendelian randomization study trauma and common psychiatric disorders
Source: Front Psychiatry. 2023 Jul 24;14:1152005. doi: 10.3389/fpsyt.2023.1152005 (PMC10406133; doi:10.3389/fpsyt.2023.1152005)
Supplement: Supplementary file 1 [file Data_Sheet_1.docx]

Supplementary materials to:

**Assessing the causal association of trauma with subsequent psychiatric disorders by a Mendelian randomization study**

Dongqing Gu, Ph.D.^1^, Shan Ou, Ph.D.^2^, Guodong Liu, Ph.D.^3^*

**Affiliations:**

^1^Department of Infectious Diseases, First Affiliated Hospital, Army Medical University, Chongqing 400038, China

^2^Department of Anesthesiology, First People’s Hospital of Chengdu, Chengdu 610016, China

^3^The Eighth Department, State Key Laboratory of Trauma, Burns and Combined Injury, Research Institute of Surgery, Daping Hospital, Army Medical University, Chongqing 400042, China

***Correspondence to:**

Guodong Liu, Ph.D.

The Eighth Department, State Key Laboratory of Trauma, Burns and Combined Injury, Research Institute of Surgery, Daping Hospital, Army Medical University,

10 Changjiang Branch Road, Yuzhong District, Chongqing 400042, China

Phone: 86-1398-3051-093

E-mail: [frankliugd@163.com](mailto:frankliugd@163.com)

Supplementary Table S1. Single nucleotide polymorphisms used as instrumental variables of trauma exposure.

| **SNP** | **CHR** | **Position** | **Effect allele** | **Beta** | **SE** | **P-value** | **Sample size** | **MAF** | **R^2^** |
| --- | --- | --- | --- | --- | --- | --- | --- | --- | --- |
| rs140746906 | 2 | 164732034 | T | 0.03 | 0.01 | 2.00E-06 | 98720 | 0.01 | 0.00023 |
| rs55984850 | 16 | 89941512 | G | 0.03 | 0.01 | 4.00E-06 | 98720 | 0.01 | 0.00022 |
| rs117659769 | 17 | 75210140 | A | 0.03 | 0.01 | 2.00E-06 | 98720 | 0.01 | 0.00023 |
| rs147967569 | 20 | 7800099 | T | 0.03 | 0.01 | 7.00E-07 | 98720 | 0.02 | 0.00025 |
| rs142592479 | 2 | 49555287 | A | 0.05 | 0.01 | 4.00E-06 | 98720 | 0.02 | 0.00022 |
| rs17676433 | 10 | 119348012 | T | 0.05 | 0.01 | 2.00E-06 | 98720 | 0.03 | 0.00023 |
| rs112201895 | 5 | 62022147 | A | 0.03 | 0.01 | 2.00E-06 | 98720 | 0.04 | 0.00023 |
| rs13336594 | 16 | 1482864 | A | 0.03 | 0.01 | 5.00E-06 | 98720 | 0.05 | 0.00021 |
| rs147542932 | 1 | 196204734 | C | 0.05 | 0.01 | 3.00E-06 | 98720 | 0.06 | 0.00022 |
| rs73207111 | 8 | 9825412 | A | 0.03 | 0.01 | 1.00E-06 | 98720 | 0.09 | 0.00024 |
| rs16972410 | 16 | 73730901 | T | 0.03 | 0.01 | 4.00E-07 | 98720 | 0.10 | 0.00026 |
| rs2405628 | 12 | 85833215 | T | 0.03 | 0.01 | 8.00E-07 | 98720 | 0.14 | 0.00025 |
| rs4978349 | 9 | 108357141 | A | 0.03 | 0.01 | 9.00E-07 | 98720 | 0.15 | 0.00024 |
| rs77660130 | 5 | 73837694 | A | 0.03 | 0.01 | 3.00E-06 | 98720 | 0.15 | 0.00022 |
| rs10068776 | 5 | 168028590 | G | 0.03 | 0.01 | 3.00E-07 | 98720 | 0.18 | 0.00027 |
| rs142356570 | 3 | 84873734 | C | 0.03 | 0.01 | 3.00E-06 | 98720 | 0.18 | 0.00022 |
| rs10507710 | 13 | 64493788 | G | 0.03 | 0.01 | 2.00E-07 | 98720 | 0.22 | 0.00027 |
| rs12682334 | 8 | 109988276 | T | 0.03 | 0.01 | 6.00E-07 | 98720 | 0.23 | 0.00025 |
| rs1957316 | 14 | 33524312 | A | 0.03 | 0.01 | 5.00E-06 | 98720 | 0.25 | 0.00021 |
| rs6959900 | 7 | 24775954 | A | 0.03 | 0.01 | 2.00E-07 | 98720 | 0.26 | 0.00027 |
| rs16896341 | 8 | 121874511 | A | 0.03 | 0.01 | 2.00E-06 | 98720 | 0.26 | 0.00023 |
| rs6445961 | 3 | 58246932 | A | 0.03 | 0.01 | 1.00E-07 | 98720 | 0.27 | 0.00029 |
| rs67526282 | 18 | 55803956 | C | 0.03 | 0.01 | 4.00E-06 | 98720 | 0.31 | 0.00022 |
| rs3754748 | 2 | 173073496 | A | 0.03 | 0.01 | 2.00E-06 | 98720 | 0.32 | 0.00023 |
| rs4705007 | 5 | 156335618 | G | 0.03 | 0.01 | 2.00E-08 | 98720 | 0.34 | 0.00032 |
| rs28740872 | 14 | 98088347 | T | 0.03 | 0.01 | 1.00E-08 | 98720 | 0.37 | 0.00033 |
| rs2381473 | 2 | 143394233 | T | 0.03 | 0.01 | 1.00E-06 | 98720 | 0.43 | 0.00024 |
| rs3120637 | 15 | 79562787 | C | 0.03 | 0.01 | 1.00E-06 | 98720 | 0.47 | 0.00024 |
| rs4765074 | 12 | 126083250 | A | 0.03 | 0.01 | 6.00E-07 | 98720 | 0.48 | 0.00025 |
| rs7213514 | 17 | 29210224 | T | 0.03 | 0.01 | 2.00E-06 | 98720 | 0.50 | 0.00023 |
| rs4074990 | 1 | 73256777 | G | 0.03 | 0.01 | 4.00E-07 | 98720 | 0.50 | 0.00026 |
| rs1476535 | 7 | 114430980 | T | 0.04 | 0.01 | 1.00E-10 | 98720 | 0.46 | 0.00042 |
| rs7231178 | 18 | 53227371 | A | 0.03 | 0.01 | 4.00E-06 | 98720 | 0.45 | 0.00022 |
| rs12614303 | 2 | 124289603 | C | 0.03 | 0.01 | 9.00E-07 | 98720 | 0.45 | 0.00024 |
| rs2410949 | 5 | 107119024 | C | 0.03 | 0.01 | 8.00E-07 | 98720 | 0.43 | 0.00025 |
| rs4312987 | 6 | 53720234 | T | 0.05 | 0.01 | 2.00E-06 | 98720 | 0.41 | 0.00023 |
| rs3896224 | 10 | 104708095 | A | 0.03 | 0.01 | 3.00E-07 | 98720 | 0.40 | 0.00027 |
| rs10468608 | 17 | 21283048 | C | 0.05 | 0.01 | 5.00E-06 | 98720 | 0.37 | 0.00021 |
| rs10968265 | 9 | 28002525 | A | 0.03 | 0.01 | 2.00E-06 | 98720 | 0.34 | 0.00023 |
| rs6227 | 15 | 90882002 | C | 0.03 | 0.01 | 2.00E-08 | 98720 | 0.34 | 0.00032 |
| rs283015 | 4 | 16727694 | C | 0.03 | 0.01 | 2.00E-06 | 98720 | 0.34 | 0.00023 |
| rs524562 | 3 | 153172818 | T | 0.03 | 0.01 | 2.00E-06 | 98720 | 0.32 | 0.00023 |
| rs4682660 | 3 | 134300524 | G | 0.03 | 0.01 | 2.00E-06 | 98720 | 0.30 | 0.00023 |
| rs1390496 | 1 | 187307421 | G | 0.05 | 0.01 | 5.00E-06 | 98720 | 0.26 | 0.00021 |
| rs7661756 | 4 | 96176288 | C | 0.03 | 0.01 | 2.00E-06 | 98720 | 0.24 | 0.00023 |
| rs66664020 | 6 | 17012781 | A | 0.03 | 0.01 | 8.00E-08 | 98720 | 0.24 | 0.00029 |
| rs11671136 | 19 | 29804726 | G | 0.03 | 0.01 | 4.00E-06 | 98720 | 0.23 | 0.00022 |
| rs74514008 | 6 | 107721176 | G | 0.03 | 0.01 | 4.00E-07 | 98720 | 0.15 | 0.00026 |
| rs4714735 | 6 | 44057962 | G | 0.03 | 0.01 | 3.00E-06 | 98720 | 0.12 | 0.00022 |
| rs1832877 | 9 | 29048442 | G | 0.03 | 0.01 | 1.00E-07 | 98720 | 0.11 | 0.00029 |
| rs111749255 | 14 | 35328673 | G | 0.03 | 0.01 | 2.00E-07 | 98720 | 0.08 | 0.00027 |
| rs79385087 | 16 | 60603143 | C | 0.03 | 0.01 | 3.00E-08 | 98720 | 0.05 | 0.00031 |
| rs35099409 | 8 | 28876569 | T | 0.05 | 0.01 | 5.00E-06 | 98720 | 0.05 | 0.00021 |
| rs35412563 | 22 | 19174844 | G | 0.03 | 0.01 | 8.00E-07 | 98720 | 0.05 | 0.00025 |
| rs13214628 | 6 | 132741777 | G | 0.05 | 0.01 | 1.00E-06 | 98720 | 0.03 | 0.00024 |
| rs138567168 | 11 | 35778692 | C | 0.05 | 0.01 | 5.00E-06 | 98720 | 0.02 | 0.00021 |
| rs73176142 | 3 | 171542960 | C | 0.05 | 0.01 | 3.00E-06 | 98720 | 0.02 | 0.00022 |
| rs113522753 | 16 | 71898534 | A | 0.03 | 0.01 | 4.00E-06 | 98720 | 0.02 | 0.00022 |
| rs139193462 | 19 | 15277406 | G | 0.03 | 0.01 | 2.00E-06 | 98720 | 0.01 | 0.00023 |
| rs182013377 | 10 | 64731271 | A | 0.03 | 0.01 | 2.00E-06 | 98720 | 0.01 | 0.00023 |

CHR: Chromosome, MAF: Minor allele frequency; SE: Standard error; SNP: Single nucleotide polymorphism.

**Supplementary Figure S1. Forest plots of the genetic causal association between trauma and seven common psychiatric disorders.** (A) depression; (B) bipolar disorder; (C) anxiety disorder; (D) sleep disorder; (E) eating disorder; (F) schizophrenia

| (A) | (B) |
| --- | --- |
| 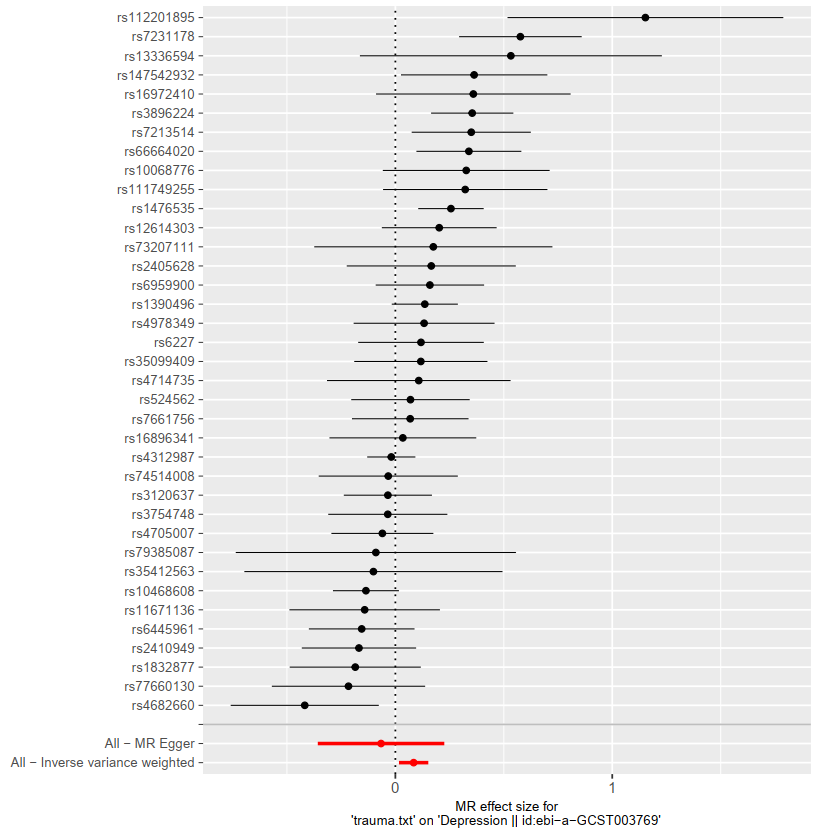 | 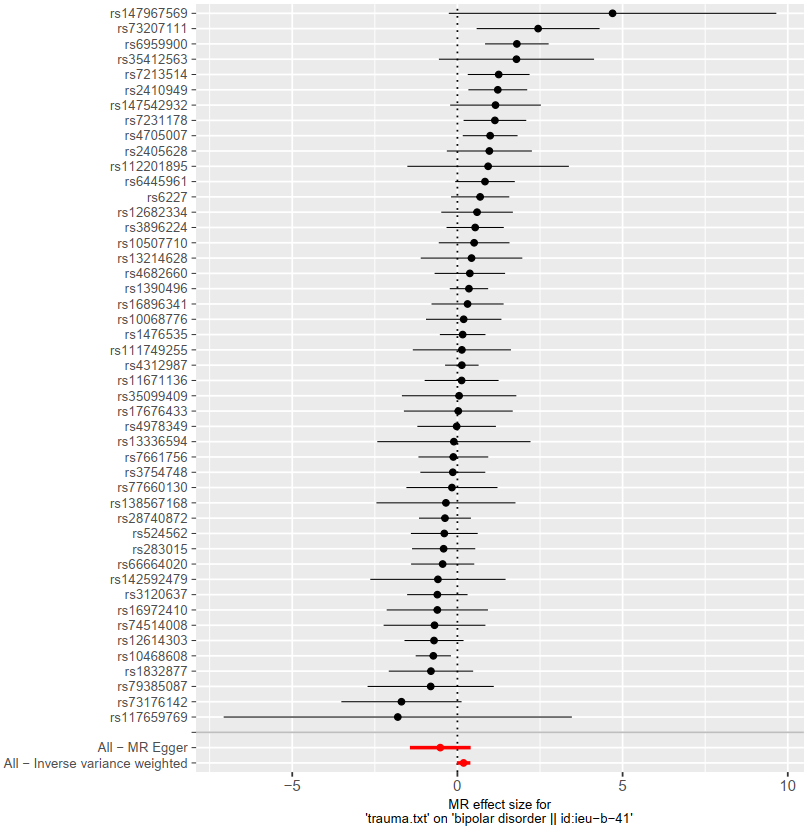 |
| (C) | (D) |
| 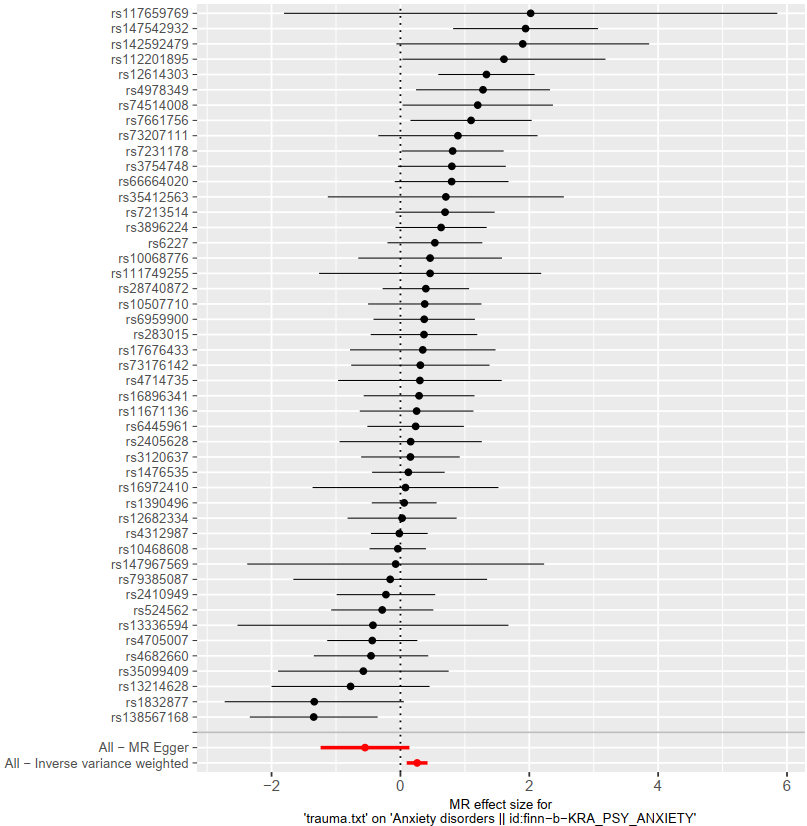 | 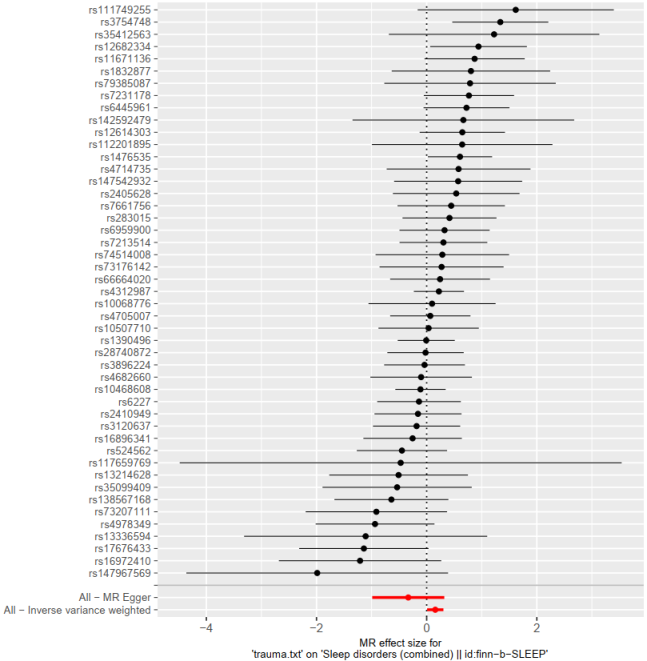 |

| (E) | (F) |
| --- | --- |
| 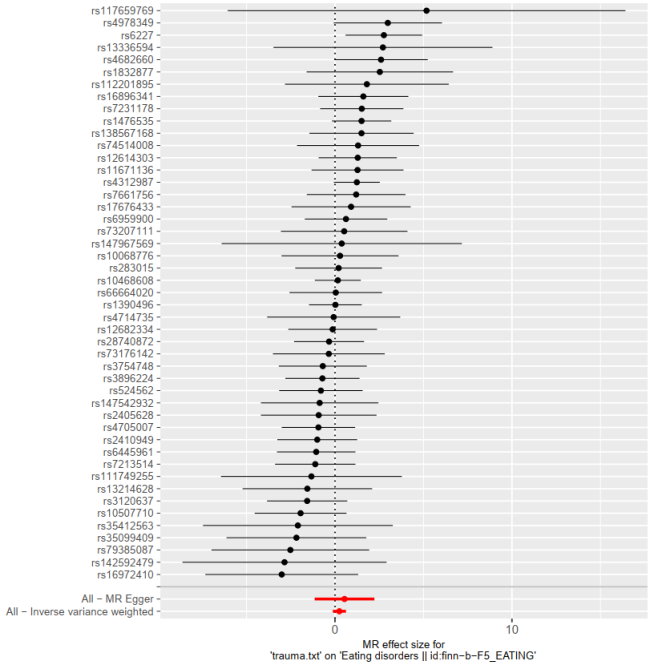 | 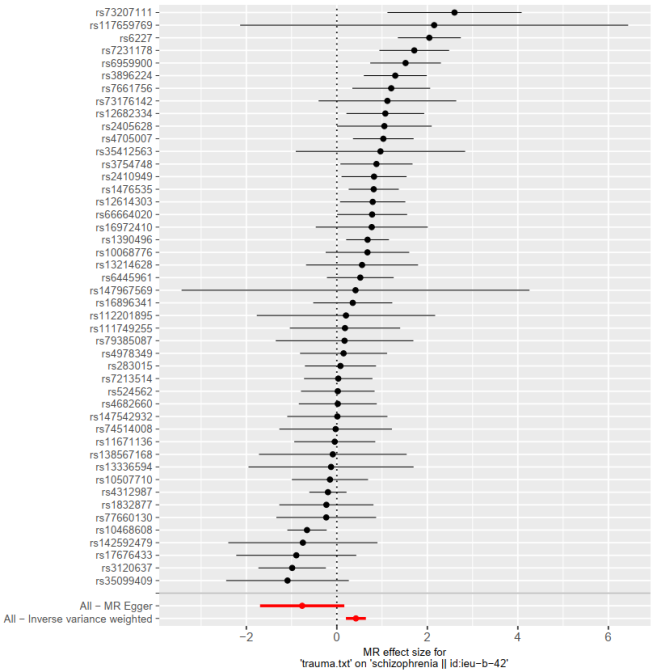 |

**Supplementary Figure S2. Scatter plots of the genetic causal association between trauma and seven common psychiatric disorders.** (A) depression; (B) bipolar disorder; (C) anxiety disorder; (D) sleep disorder; (E) eating disorder; (F) schizophrenia

| (A) | (B) |
| --- | --- |
| 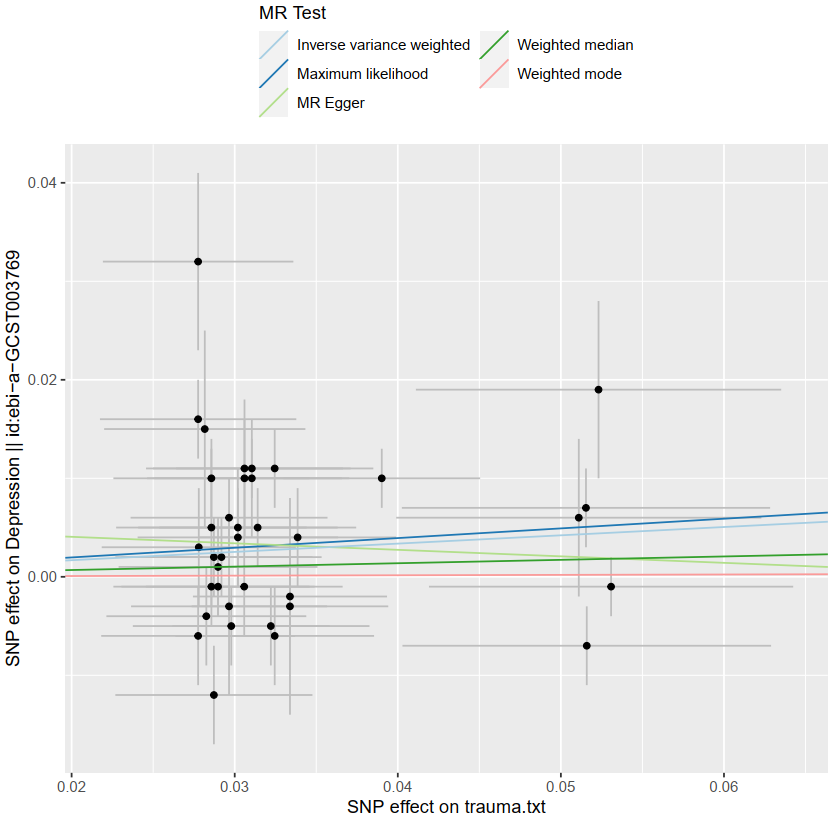 | 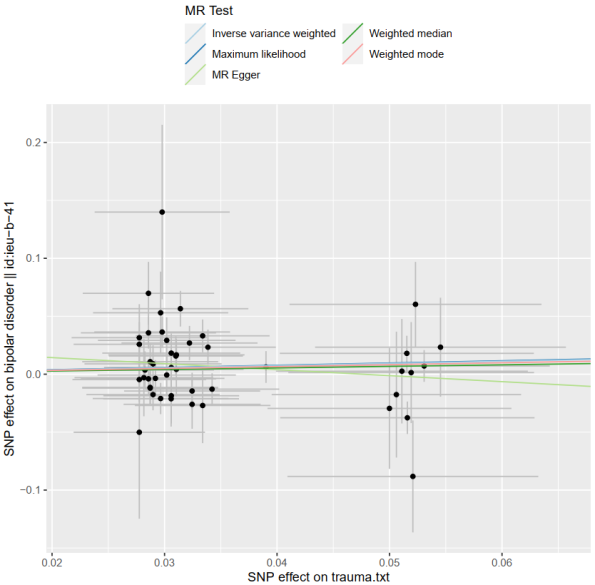 |
| (C) | (D) |
| 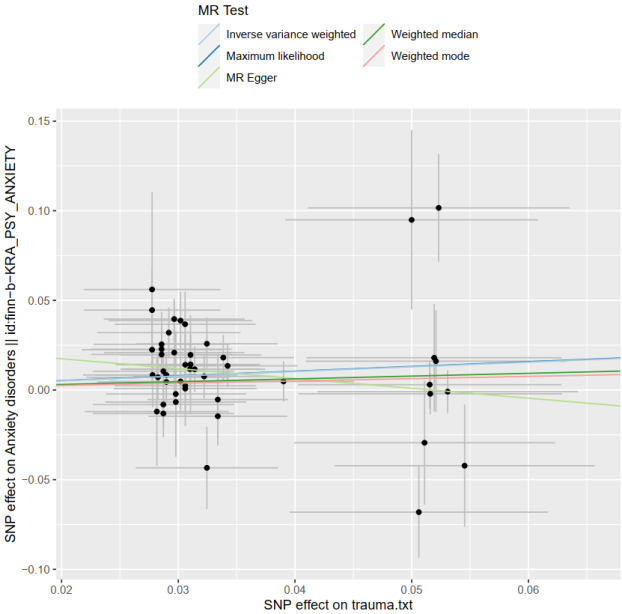 | 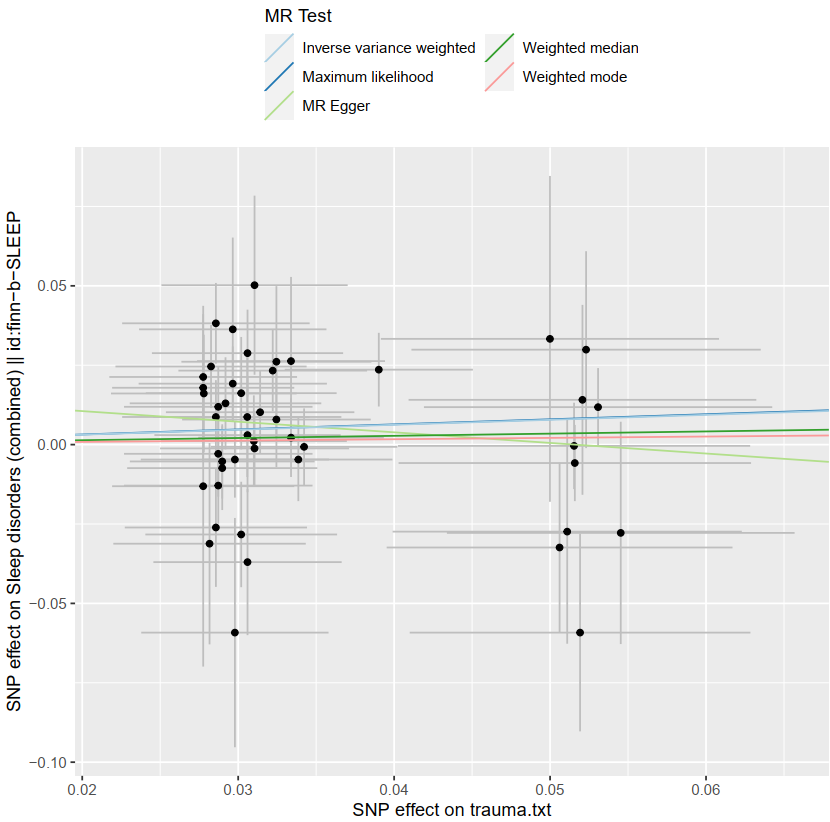 |

| (E) | (F) |
| --- | --- |
| 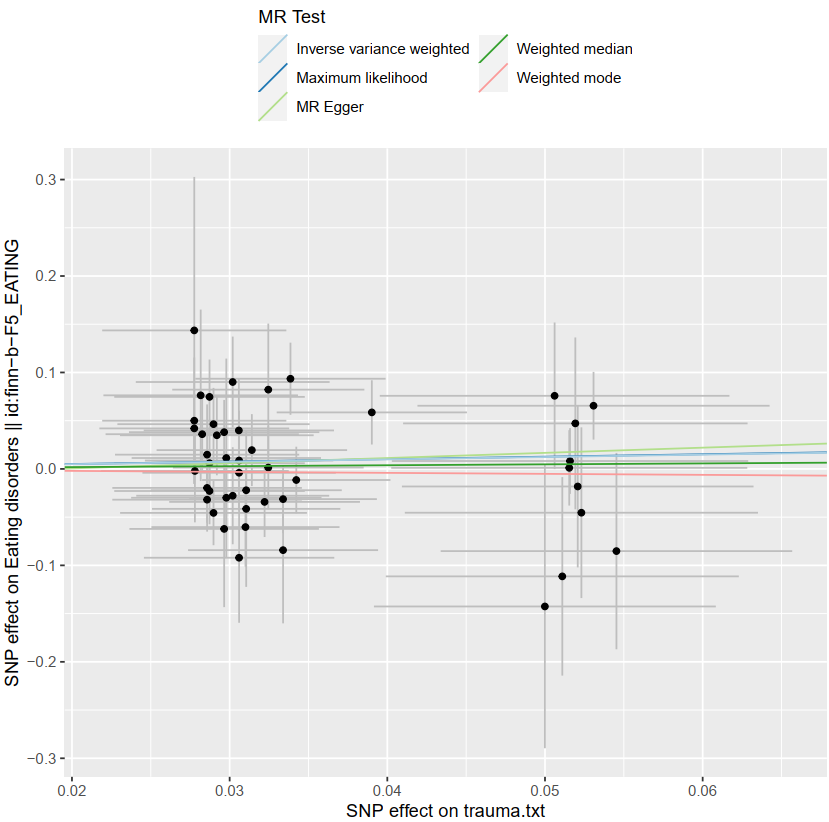 | 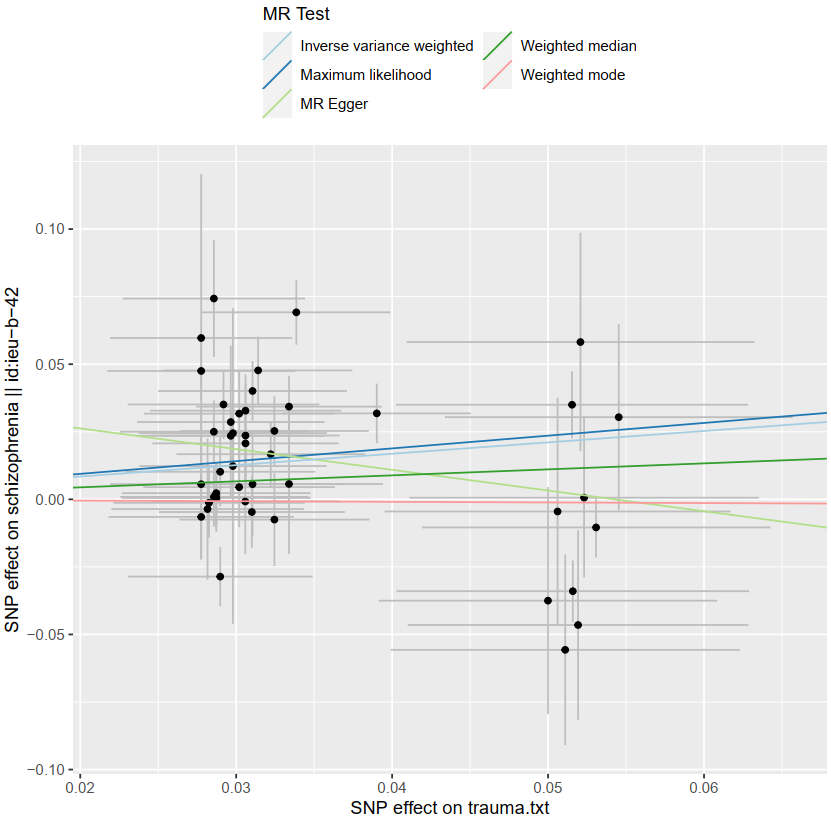 |

**Supplementary Figure S3. Leave-one-out plots of the genetic causal association between trauma and seven common psychiatric disorders.** (A) depression; (B) bipolar disorder; (C) anxiety disorder; (D) sleep disorder; (E) eating disorder; (F) schizophrenia

| (A) | (B) |
| --- | --- |
| 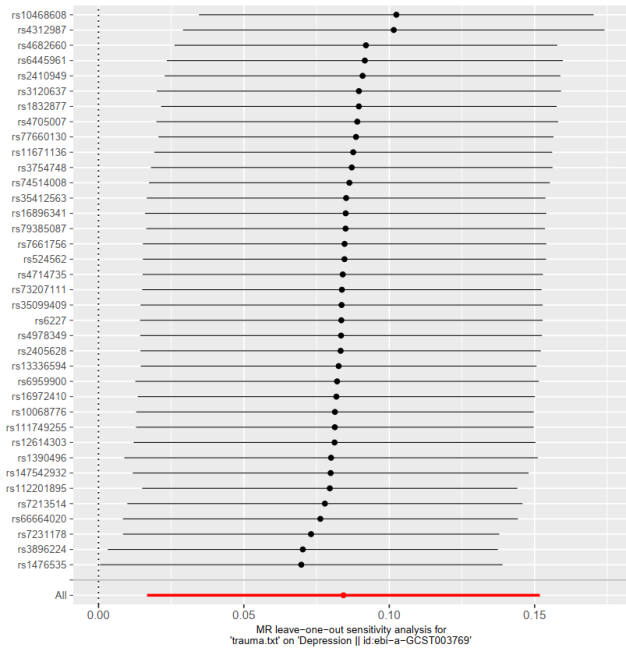 | 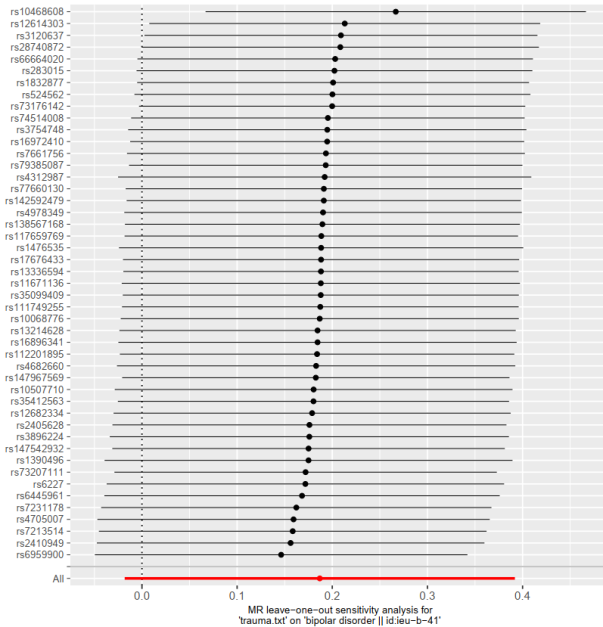 |
| (C) | (D) |
| 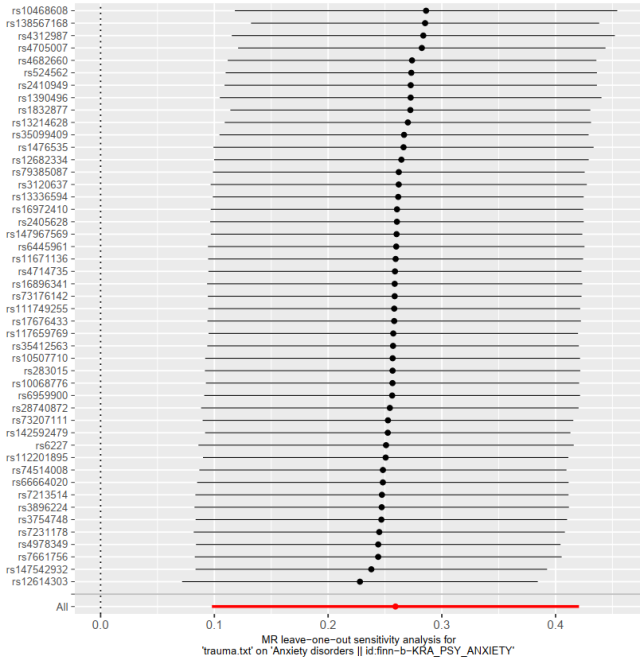 | 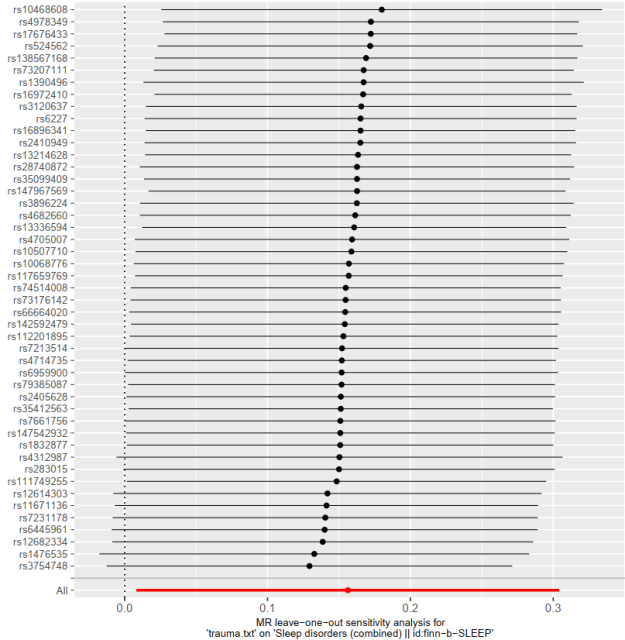 |

| (E) | (F) |
| --- | --- |
| 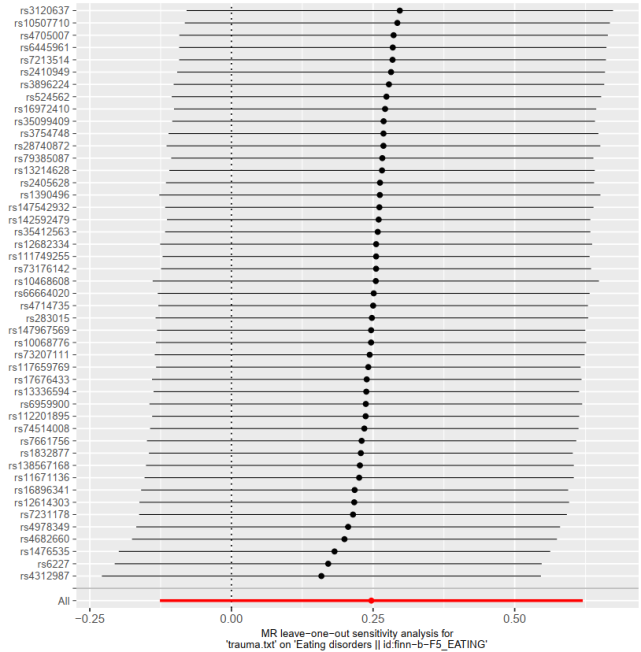 | 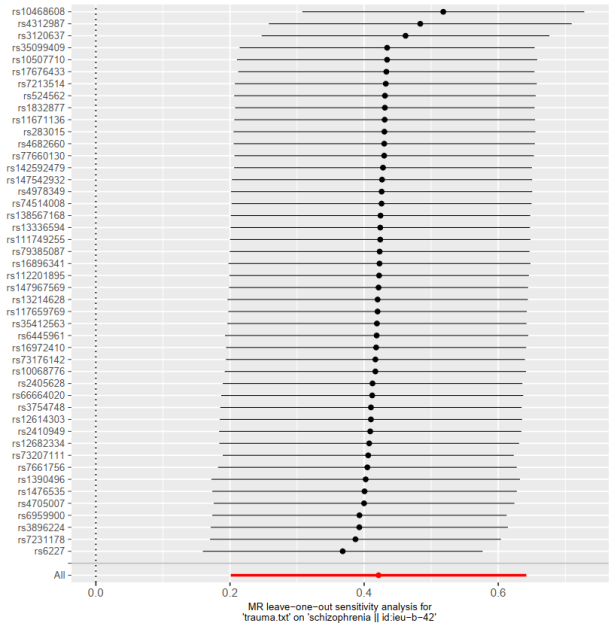 |

**Supplementary Figure S4.** **Funnel plots of the genetic causal association between trauma and seven common psychiatric disorders.** (A) depression; (B) bipolar disorder; (C) anxiety disorder; (D) sleep disorder; (E) eating disorder; (F) schizophrenia

| (A) | (B) |
| --- | --- |
| 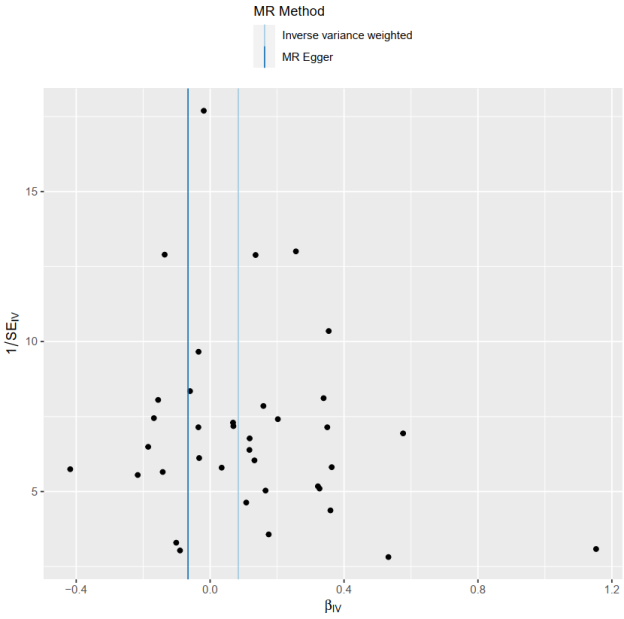 | 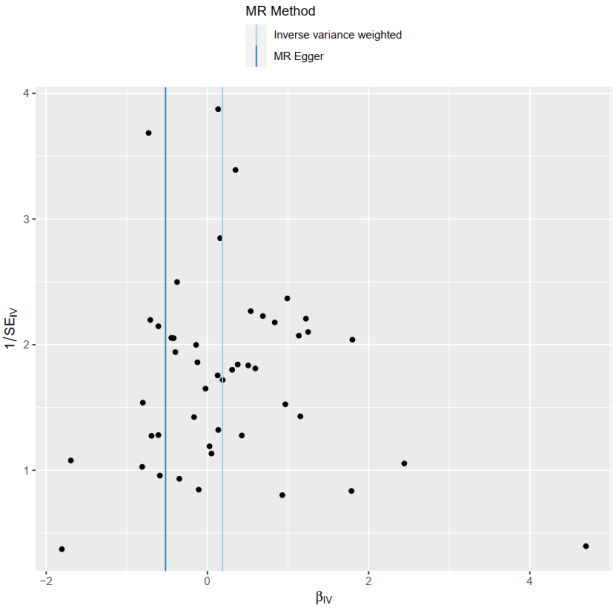 |
| (C) | (D) |
| 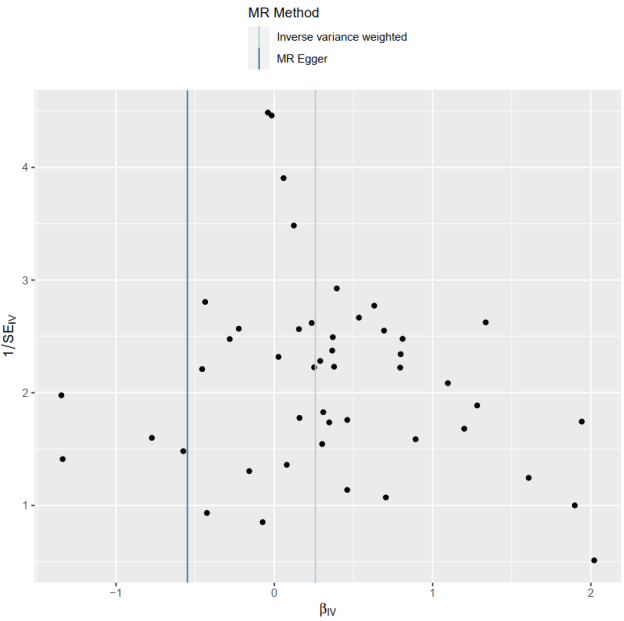 | 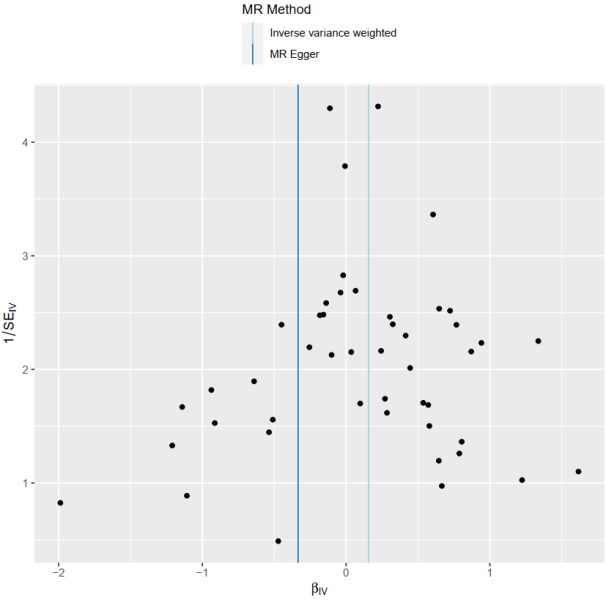 |

| (E) | (F) |
| --- | --- |
| 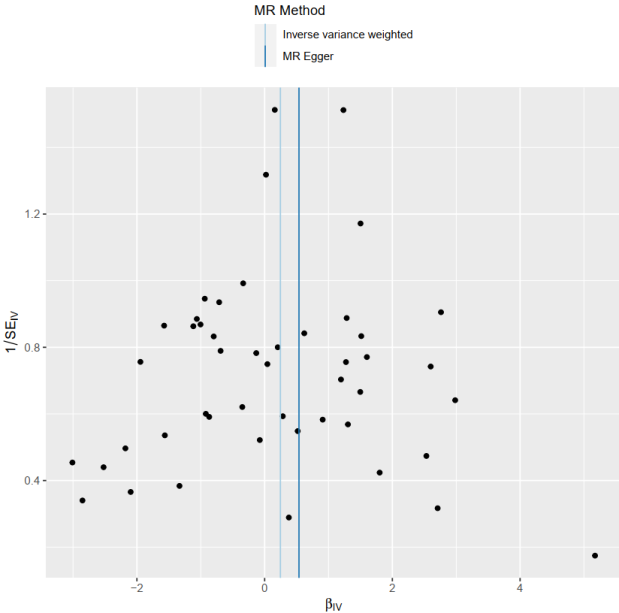 | 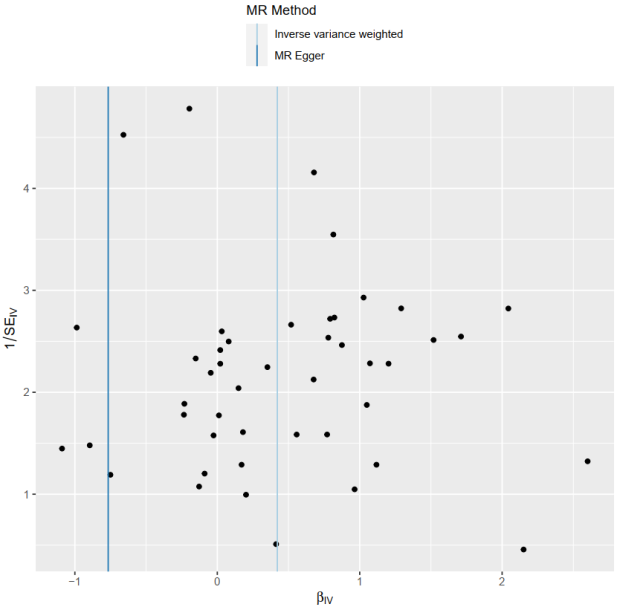 |
